# Association between body mass index and health outcomes among adolescents: the mediating role of traditional and cyber bullying victimization

**DOI:** 10.1186/s12889-018-5390-0

**Published:** 2018-05-30

**Authors:** Byung Lee, Seokjin Jeong, Myunghoon Roh

**Affiliations:** 1 Central Connecticut State University, 1615 Stanley Street, New Britain, CT, USA; 2 University of Texas at Arlington, 701 S. Nedderman Drive, Arlington, TX 76019 USA; 3 Texas A&M University - San Antonio, One University way, San Antonio, TX 78224 USA

**Keywords:** Obesity, Body mass index, Physical distress, Psychological distress, Traditional bullying victimization, Cyberbullying victimization, Weight-based victimization

## Abstract

**Background:**

It is well-documented that obese children and adolescents tend to experience a variety of negative physical and psychological health consequences. Despite the association between obesity and physical and psychological well-being, few studies have examined the role of off-line and on-line forms of bullying victimization in this link. The main objective of the current study is to investigate the direct and mediating effects of traditional and cyber bullying victimization in explaining the relationship between the body mass index (BMI) and physical/psychological distress.

**Methods:**

A nationally representative sample of 10,160 school children (mean age = 12.95 ± 1.75) were collected from the 2009 Health Behavior in School-aged Children (HBSC) study. Data were collected on body mass index, physical and psychological health, bullying victimization experience, and demographic information. A seemingly unrelated regression (SUR) was employed to assess and compare the indirect effects in multiple mediation models.

**Results:**

While a significant direct association was found between BMI and both physical and psychological health, the indirect effect of BMI on physical distress was significant only via traditional bullying victimization. Both forms of bullying victimization had a mediating impact between BMI and psychological distress. However, the indirect effect on psychological distress was manifested through a negative mediating role of cyberbullying victimization. The negative relation between cyberbullying victimization and psychological distress warrants further exploration.

**Conclusions:**

Obesity represents a serious risk to adolescent health and well-being, both physically and psychologically. If becoming a victim of traditional bullying mediates (specifically exacerbates) the level of physical and psychological distress among obese and overweight adolescents, health professionals need to focus on raising awareness of the importance of weight-based victimization for children and adolescents with obesity. School administrators and teachers could increase the efforts to identify school-age children who are stigmatized for their weight and recommend coping strategies for distressed victims of traditional and cyberbullying.

## Background

Obesity is one of the leading public health concerns in the United States, presenting a considerable threat to the well-being and health of school-aged youth. Recent statistics illustrate that obesity rate remains high among children and adolescents: while about 1 in 5 reported to be obese or overweight based on the body mass index (BMI), hereafter referred to as BMI, those aged from 12 to 19 years with extreme obesity increased to slightly over 9% during the past two decades [1]. Such prevalence of obesity may lead to deleterious health problems, physically [2, 3] and psychosocially [4–6].

Obesity in childhood and adolescence has been linked to a wide array of physical health outcomes. Specifically, obesity-related physical health symptoms include, but not limited to, headaches, stomachaches, somatic complaints, sleep difficulties, and school/social functioning [7–9]. School children and adolescents with obesity also suffer from psychological and emotional problems such as depression [10], anxiety [11], low self-esteem [12], and lack of emotional support and cognitive stimulation [13].

Further, obesity during childhood and adolescence has been shown to be stigmatizing and likely to result in social adversity. There is a strong bias and prejudice towards school children with obesity [14]. Obese children are perceived as the least favorable classmates by their peers in school [15] and often labeled with various negative stereotypes [16]. The weight-based prejudice from peers may be formed as early as three years of age [17]. Upon entering elementary school, obese and overweight youth are likely to experience weight-related judgement and social outcomes such as rejection from peers or loss of friends [18–20]. In addition, research indicates that stigma and attitude based on weight bias could originate from educators such as teachers [21, 22] or parents and siblings [23].

The weight-based stigma and hostility is also pervasive in the on-line domain. A qualitative analysis of social media content (e.g. Twitter) illustrates a prominent theme of offensive and prejudiced attitude and perception towards the notion of obesity [24]. Among a wide range of stigmatizing content, obese individuals are perceived largely as gluttonous, unattractive, and sedentary [25]. Based on a person’s weight or body size, youthful victims are stereotyped in a discriminatory, biased manner.

In addition to biased perceptions of one’s weight, prior research suggests that being obese or overweight contributes to the likelihood of becoming a victim of traditional bullying [26]. Prior studies examining the impact of BMI on peer victimization found that overweight and obese children were more likely to be victimized by specific forms of bullying (verbal, relational, physical) compared to those with normal weight [27, 28]. A related study, based on reports from teachers, mothers, and student themselves, showed a significant relationship between being obese and the odds of being bullied among sixth grade children, after controlling for sociodemographic characteristics [19]. Given the widespread bias towards obesity, overweight children were more likely to experience weight-specific teasing perpetrated by peers in general compared to non-overweight children [29].

More specifically, children have witnessed their overweight or obese peers to experience teasing in public area and during physical activities, exclusion from social activities, spreading of negative rumors, verbal threats and physical harassment [30–32]. This appears to be consistent across gender; both obese boys and girls had a higher probability of becoming victims of overt forms of bullying (e.g. hitting, shoving, name-calling) than their average weight peers [33]. However, other research has revealed that while females were primarily victims of verbal and relational bullying, males were more likely to be victims of all types (including verbal, physical, social exclusion, rumor spreading, and cyber bullying) [34].

Despite only a few empirical findings, the stigmatization of being obese or overweight and risk for peer victimization are evident in cyberspace. In a study using a sample of school adolescents seeking weight loss treatment, more than half of the participants reported that they experienced weight-based cyberbullying victimization via computers or cell phones [28]. While 61% of these youth have encountered on-line posting of embarrassing content, 59% have received mean text messages, e-mails, or instant messages. According to a more recent study of patients in residential facilities for severe obesity, obese adolescents were significantly more likely to be bullied via the Internet compared to their normally weighted peers [29]. Furthermore, body dissatisfaction is correlated with cyberbullying victimization; youth who are victims of cyberbullying are twice as likely to perceive one’s body to be ‘too fat’ compared to those who have not been victimized [30]. Given the stigma and bias associated with obesity and the greater visibility of offensive comments or images via social media [25, 31, 32], weight-based victimization in online settings can be detrimental to the psychological and physical health of adolescents.

Prior studies have shown that adverse outcomes and responses are associated with weight-based victimization among school children. While adolescents who have been victims of weight-based teasing or bullying tend to feel depressed, sad, angry, afraid, and dissatisfied with their body, some are more likely to have negative reactions in and outside school such as avoidance strategies, binge eating, skipping schools, poor academic performance in the event of teasing or bullying by peers [33]. Traditional bullying victimization is found to be associated with poor physical health, including somatic symptoms and withdrawn behaviors [34, 35]. Similarly, cyberbullying negatively impacts the emotional and psychosocial well-being of those who are victimized. Specifically, victims of cyberbullying can suffer from social anxiety [36], depressive symptoms [37], decreased self-esteem [38], suicidal thoughts [39], emotional distress [40], sadness [41], and angry feelings [42]. Moreover, being victimized on-line undermines one’s academic performance in school [43], and further triggers problematic behaviors such as truancy [38], alcohol use and weapon carrying [44]. Furthermore, obese adolescents who have been victims of cyberbullying showed a higher level of suicidal ideation compared to their peers with normal weight [29].

Notwithstanding findings indicating a strong relationship between bullying victimization and physical or psychological health, only a handful of studies have examined the longitudinal relationship between these factors. Involvement in traditional and cyber forms of bullying was found to be related to mental health and psychosocial problems such as depressive and emotional symptoms, social anxiety, ADHD symptoms, and lower levels of well-being [45–50]. Similarly, minimal attention has been devoted to the longitudinal investigation of obesity and overweight with bullying behaviors. While a significant association between childhood obesity and the likelihood of being bullied was observed [19] among sixth grade children, bullying victimization during adolescence was linked to an increased risk of obesity and higher BMI when reaching young adulthood [51, 52]. The question emerging from these longitudinal findings concerns whether bullying victimization could be mediating the relationship between weight status and physical and psychological outcomes.

To date, no studies have examined the mediating role of bullying victimization in the relationship between obesity and both physical and psychological distress. Considering that adolescent obesity is correlated with a greater likelihood of being victimized, understanding the effect of victimization, both off-line and on-line, on one’s level of physical and psychological distress would be of particular value in developing treatments for victims to cope with their distress. The current study addresses the following research questions.

1. To what extent do overweight and obese youth experience traditional and cyberbullying victimization, compared to normal weight youth?
2. To what extent do overweight and obese victims of traditional or cyberbullying experience physical and psychological distress compared to normal weight youth?
3. Does becoming a victim of either traditional or cyberbullying mediate the relationship between BMI and physical / psychological distress?

## Methods

### Data collection

Data used in the current study was collected from the 2009 U.S. version of the Health Behavior in School-Aged Children (HBSC) survey as the key source of our analysis. This nationally representative data, collected from 42 countries in collaboration with the World Health Organization, provides detailed information on health- and school violence-related behaviors [53]. Of the respondents, the mean age was 12.9, whereas 51.4% were boys and 48.8% were white. It must be noted that some of the variables in our analysis had 4 to 5% of missing responses. Missing values must be properly dealt with due to the fact that improper handling could yield biased coefficients [54]. Following an analysis of missing data, the results confirmed that the missing observations for most of the key measures under study are missing not at random. Instead of a multiple imputation to generate probable responses, cases with missing data were listwise deleted. Among the 12,642 respondents who completed the survey through multi-stage sampling, 2482 were excluded based on missing information. The final sample yielded 10,160 children.

### Measures

#### Physical and psychological distress

We focused on two health related measures as outcomes: i) *physical distress* and ii) *psychological distress*. The physical distress scale (α = .65) consists of three items that measure the extent of various pain related physical conditions in the last six months: (1) “How often have you had the headaches,” (2) “How often have you had the stomachaches,” and (3) “How often have you had the backaches.” Additionally, the psychological distress scale (α = .75) was created by summing five items that reflect respondents’ psychological health in the last six months. These items are: (1) “How often have you had the feeling low?” (2) “How often have you had the irritability or bad temper?” (3) “How often have you had the feeling nervous?” (4) “How often have you had the difficulties in getting to sleep?” and (5) “How often have you had the feeling dizzy?” Response options for each of these items ranged from 0 (rarely or never) to 4 (about every day) during the last six months. Both physical and psychological distress scales were coded so that a higher score indicates a lower level of physical / psychological distress in the last six months.

#### Traditional and cyber bullying victimization (*mediator variables*)

The current study examines the indirect effects of obesity on health-related outcomes by investigating the mediating influence of traditional and cyberbullying victimization. First, *traditional bullying victimization* is a seven-item measure (α = .93) that assesses the aspects of physical and emotional victimization. These measures were adopted from the previous [55]. Respondents indicated how often they have been bullied at school during the past couple months: (1) “I was called mean names, was made fun of, or teased in a hurtful way,” (2) “Other students left me out of things on purpose, excluded me from their group of friends, or completely,” (3) “I was hit, kicked, pushed, shoved around, or locked indoors,” (4) “Other students told lies or spread false rumors about me and tried to make others dislike me,” (5) “I was bullied with mean names and comments about my race or color,” (6) “I was bullied with mean names and comments about my religion,” and (7) “Other students made sexual jokes, comments or gestures to me.” The traditional bullying victimization scale was created using the items above and coded so that a higher score indicates a higher frequency of victimization at school. Second, *cyberbullying victimization* was a four-item measure (α = .90) assessing an individual’s victimization experience using a computer-mediated communication. These measures were adopted from a study of Olweus [56]. Respondents were asked to indicate how frequently they have been bullied during the past couple months: (1) “I was bullied at school using a computer or e-mail messages or pictures,” (2) “I was bullied at school using a cell phone,” (3) “I was bullied outside of school using a computer or e-mail messages or pictures,” and (4) “I was bullied outside of school using a cell phone.” Each measure of victimization was based on a five-point Likert scale response ranging from (1) none during the past several months to (5) several times a week. For the purpose of the current study, the cyberbullying victimization scale was created by summing four items and coded so that a higher score indicates a more frequent victimization via the Internet.^1^

#### Body mass index (BMI)

As an indicator of obesity, *body mass index (BMI)* was computed based on self-reported measures of height and weight for each respondent [55]. The BMI percentiles were computed based on the formula [Weight(lbs)/[Height(inches)*Heights(inches)] * 703. Given that the formula was mainly aimed to compute the adult BMI, BMI percentiles were calculated by taking into account the respondent’s gender and age for accurate interpretation. BMI percentiles were then coded into four categories based on the criteria established by the Center for Disease Control: (1) underweight – less than 5th percentile; (2) healthy weight – between 5th and 85th percentile; (3) at risk of overweight – between 85th and 95th percentile; and (4) overweight – greater than 95th percentile. For the current analyses, the healthy weight between 5th and 85th percentile was used as a reference category to explore the effect of overweight and obesity.^2^

#### School-related and demographic characteristics

As demonstrated by prior research that social-demographic characteristics are significant indicators of bullying victimization and school-life related factors, we also incorporated demographic variables as control variables. *Gender* (male = 1), *age* (in years), ethnicity (Hispanic = 1), and *race* (White = 1) were included in this study. The five categories – African-American (17.1%), Asian (3.7%), American Indian or Alaska Native (1.8%), Native Hawaiian or Other Pacific Islander (0.9%), two or more races (6.5%), and other (18.9%) – were collapsed into non-White. Mean scores, standard deviations, and ranges for all variables are presented in Table 1.

**Table 1:** Descriptive Statistics of Study Variables (*n* = 10,160)

Variable	Range or Frequency	Mean or Percentage	Standard Deviation
Dependent Variables			
*Physical Distress*	0~12^a^	2.95^a^	2.87^a^
*Psychological Distress*	0~20^a^	5.39^a^	4.75^a^
Independent Variables			
* BMI (Body Mass Index)* (%)			
Healthy Weight	6465^b^	63.63^b^	
Underweight	432^b^	4.25^b^	
Overweight	1855^b^	18.26^b^	
Obese	1408^b^	13.86^b^	
Mediate Variables			
*Traditional Victimization*	0~28^a^	2.69^a^	4.79^a^
*Cyber Victimization*	0~16^a^	.58^a^	2.18^a^
Control Variables			
*Gender* (1=Male) (%)	5227^b^	51.45^b^	
*Age*	10~17^a^	12.95^a^	1.75^a^
*Ethnicity* (1=Hispanic) (%)	2916^b^	28.70^b^	
*Race* (1=White) (%)	4961^b^	48.83^b^	

Note. ^a^ The range, mean, and standard deviation are reported

^b^The frequency and percentage are reported

#### Statistical analysis

The overarching aim of this study was to explore the impact of overweight and obesity on physical and psychological distress and whether these weight-based effects occur indirectly through traditional and cyberbullying victimization. A seemingly unrelated regression (SUR) was used to simultaneously assess and compare the mediating effects of two types of bullying victimization in the link between obesity and both physical and psychological distress. The aforementioned relationships will be empirically tested using multiple mediator models [57]. Since independent variables differ from one equation to the next, the use of SUR, allowing to compare multiple equations simultaneously, ensures statistical efficiency in the current research [58]. Furthermore, SUR is well suited for estimating and comparing indirect effects in multiple categories (i.e., BMI categories) [59].

Specifically, we estimated four sets of models: (1) the effect of BMI on the outcome measures (i.e., physical and psychological distress); (2) the effect of BMI on the mediators (i.e., traditional and cyber bullying victimization); (3) the effect of the mediators on outcome measures; and (4) the indirect effects of BMI on outcome measures. Additionally, Sobel-Goodman tests was applied to statistically test for the presence of mediation. To generate standard errors and significance levels of the indirect effects, we utilized 1000 bootstrap replications. All analyses were performed using *Stata SE 13* to estimate our seemingly unrelated models. Although the effects of BMI on bullying victimization and negative consequences of bullying victimizations are well-documented, very few studies to date have investigated the mediating effects of bullying victimization (traditional and cyber) among school children with obesity in the context of distress.

#### Additional analysis

To simultaneously examine the empirical relationships among the variables under investigation, structural equation modeling (SEM) was also employed by using maximum-likelihood-estimation in Stata 13.1 [60]. We carefully examined a number of different model fit indices to assess identification and stability. Since chi-square statistics assessing the fit between the matrix of observations and the matrix generated by the model is sensitively influenced by large sample size [61], we paid less attention. Instead, we considered: standardized root-mean-square residual (SRMR); root-mean-square-error of approximation (RMSEA) with 90% confidence interval [62]; and comparative fit index (CFI) [63]. For study criterion, the combination of SRMR < .08; RMSEA < .08 with *p*-value > .05; and CFI > .95 were used to identify a satisfactory or acceptable model fit. After we set all structural paths with covariance and mediators, the fit for this model was: SRMR = .013; RMSEA = .036 (p-value = .85; 90% C.I. = .030~.043); CFI = .990. The examination of model fit indices suggested that the model fitted the data well.

## Results

### Descriptives

We report summary statistics for physical and psychological distress, traditional and cyber bullying victimization, and other control variables used in the current study (See Table 1). The mean physical distress score was 2.95 (sd = 2.87) and the mean psychological distress score was 5.39 (sd = 4.75). Among the study participants in our analytic sample, 63.6% (*n* = 6465) were categorized as healthy weight, 4.3% (*n* = 432) as underweight, 18.3% (*n* = 1855) as overweight, and 13.9% (*n* = 1408) as obese. With regards to bullying victimization, the mean score for traditional victimization score was 2.69 (sd = 4.79), and the mean score for cyber victimization was .58 (sd = 2.18). However, these two mean scores reflected a relatively lower degree of victimization compared to other studies. Concerning demographic covariates, the final sample of 10,160 students showed a mean age of 12.95 years (sd = 1.75). While the majority of the sampled students were non-Hispanics (71.3%), males accounted for 51.5%.

### Bivariate correlations

As a preliminary analysis, we conducted a bivariate correlation analysis (See Table 2). As expected, BMI was significantly related to both physical and psychological distress (*r* = .06 and .06, respectively). Additionally, traditional bullying victimization and cyber bullying victimization were negatively correlated with physical distress (*r* = .22 and .12, respectively) and psychological distress (*r* = .29 and .13, respectively). Notably, BMI was positively related to both traditional and cyber victimization (*r* = .06 and .02, respectively).

**Table 2:** Correlations of Covariates (n = 10,160)

	1	2	3	4	5	6	7	8	9
Dependent Variables									
1. *Physical Distress*	1								
2. *Psychological Distress*	.62***	1							
Independent Variables									
3. *BMI (Body Mass Index)*	.06***	.06***	1						
Mediate Variables									
4. *Traditional Victimization*	.22***	.29***	.06***	1					
5. *Cyber Victimization*	.12***	.13***	.02**	.61***	1				
Control Variables									
6. *Gender* (1=Male) (%)	−.16***	−.15***	.08***	−.01	−.01	1			
7. *Age*	.11***	.10***	−.05***	−.06***	.02***	.04***	1		
8. *Ethnicity* (1=Hispanic)	−.05***	−.02	.07***	.02	.04***	.01	−.01	1	
9. *Race* (1=White)	.05***	−.01	−.09***	−.04***	−.04***	.02	−.03***	−.41***	1

Note. **p < .05. ***p < .01

### Hypothesized mediation models

#### Impact of BMI on victimization and distress

Table 3 indicates the main direct effects of BMI on mediator variables (i.e., traditional victimization and cyber victimization) and outcome variables (i.e., physical distress and psychological distress) using seemingly unrelated regression models. First, we examined whether weight status predicts the probability of bullying victimization. The results indicated that overweight children have a higher risk than healthy weight children of becoming a victim of traditional bullying (*b* = .47, *p* < .01). Similarly, obesity was positively associated with risk of being a victim of traditional bullying (*b* = .70, p < .01). Next, we examined whether weight status predicts physical and psychological distress. The results revealed that obese and overweight children have been shown to have poorer physical and psychological distress than healthy weight children (*b* = .27 and .38, *p* < .01, respectively). Moreover, obesity has been found to be positively associated with physical and psychological distress (*b* = .56 and .67, *p* < .01, respectively). Finally, traditional bullying victimization was positively associated with both physical and psychological distress (*b* = .15 and .36, p < .01, respectively).

**Table 3:** Direct Effects SUR (n = 10,160)

	Model 1			
	*Traditional Victimization*	*Cyber Victimization*	*Physical Distress*	*Psychological Distress*
	*b* (S.E.)	*b* (S.E.)	*b* (S.E.)	*b* (S.E.)
Independent Variables				
*BMI (Body Mass Index)*^*a*^				
Underweight	.50(.24)**	−.01(.11)	−.17(.15)	.23(.25)
Overweight	.47(.12)***	.06(.05)	.27(.08)***	.38(.13)***
Obese	.70(.14)***	.11(.06)	.56(.09)***	.67(.14)***
Mediate Variables				
*Traditional Victimization*			.15(.01)***	.36(.01)***
*Cyber Victimization*			−.02(.02)	−.17(03)***
Control Variables				
*Gender* (1=Male)			−1.10(.06)***	−1.76(.09)***
*Age*			.23(.02)***	.33(.03)***
*Ethnicity* (1=Hispanic)			−.12(.07)	.08(.12)
*Race* (1=White)			.34(.06)***	.18(.10)

Note. a. Healthy weight is the reference category

**p < .05. ***p < .01

#### Mediating effect of bullying victimization

The SUR estimations with traditional and cyber bullying victimization as mediators are presented in Table 4. The mediation results showed a significant indirect effect of the overweight (*b* = .07, p < .01) and obesity (*b* = .10, p < .01) on physical distress through traditional bullying victimization, but not through cyberbullying victimization. For the psychological distress, we did see a significant indirect effect of the overweight (*b* = .17, *p* < .01) and obesity (*b* = .27, p < .01) on psychological distress through traditional bullying victimization. Unexpectedly, while there was no indirect effect of the weight status on physical distress through cyberbullying victimization, we found a mediating effect (*b* = −.02, *p* < .05) for psychological distress. As shown in Table 4, the significance of the indirect effects of traditional and cyber bullying victimization were examined using Sobel-Goodman tests [64]. These tests indicated that the indirect effects linking weight status with physical distress through traditional bullying victimization were significant (z = 5.90, *p* < .01), accounting for 24% of the effect of weight status on physical distress. For psychological distress, Sobel-Goodman tests also indicated that the indirect paths linking weight status with psychological distress via traditional bullying victimization were significant (z = 6.13, p < .01), accounting for 36% of the effect of weight status on psychological distress.

**Table 4:** Direct and Indirect Effects Comparisons SUR (n = 10,160)

	*Physical Distress*				*Psychological Distress*			
	*b*	S.E.	z	Sobel z (% of total effect)	*b*	S.E.	z	Sobel z (% of total effect)
Direct Effects								
* BMI (Body Mass Index)* ^*a*^								
Underweight	−.17	.15	−1.15		.23	.25	.93	
Overweight	.27***	.08	3.61		.38***	.13	2.99	
Obese	.56***	.09	6.56		.67***	.14	4.76	
* Bullying Victimization*								
Traditional	.15***	.01	18.32		.36***	.01	27.53	
Cyber	.02	.02	−1.05		−.17***	.03	−5.66	
Indirect Effects								
*BMI on Distress through Traditional Victimization* ^*a*^				5.90*** (24%)				6.13*** (36%)
Underweight	.08**	.04	2.03		.15	.09	1.62	
Overweight	.07***	.02	3.72		.17***	.05	3.67	
Obese	.10***	.02	4.80		.27***	.05	5.15	
*BMI on Distress through Cyber Victimization* ^*a*^				2.33** (5%)				2.49** (7%)
Underweight	.01	.01	.06		.01	.02	.40	
Overweight	−.01	.01	−.75		−.01	.01	−.87	
Obese	−.01	.01	−.90		−.02**	.01	−1.99	

Note. a. Healthy weight is the reference category

**p < .05. ***p < .01

#### SEM results

Following the initial SUR analysis and inspection of the model fit indices for our models, we used SEM to examine the direct relationship between BMI, physical distress, psychological distress, and two mediators. Similar to the SUR analysis, the SEM results indicated that significant correlations were in the same direction. After the initial direct effect analysis, we examined the indirect effects -- whether BMI leads to psychological and physical distress through increases in the risk of traditional and cyber bullying victimization. Consistent with the SUR mediation models, all of the estimates were in the same direction and of similar magnitude.

**Fig. 1:**
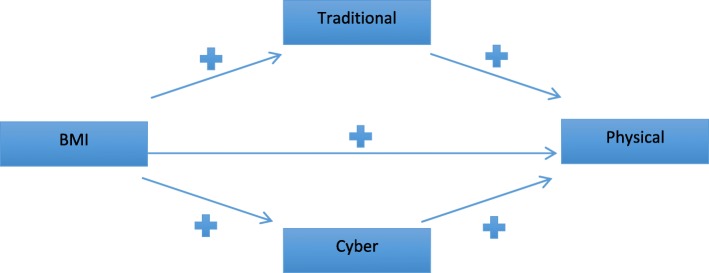
Traditional/Cyber Victimization Mediators of Obesity and Physical Distress

**Fig. 2:**
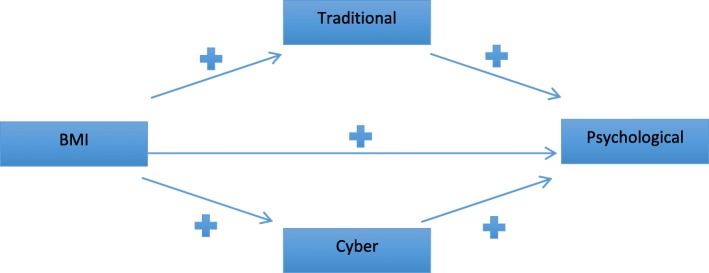
Traditional/Cyber Victimization Mediators of Obesity and Psychological Distress

**Fig. 3:**
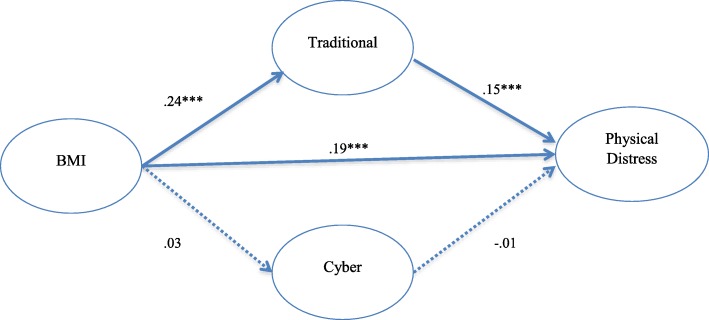
SEM: Direct and Indirect Effects of BMI on Physical Distress; The figure shows that the proportion of total “BMI” effect mediated via “Traditional” and “Cyber” Victimization was .16. Circles represent observed variables, and straight arrows connect the observed variables. Bold lines represent significant paths, and dotted lines represent nonsignificant paths. All significant parameters are significant at the *p* < .001 level

**Fig. 4:**
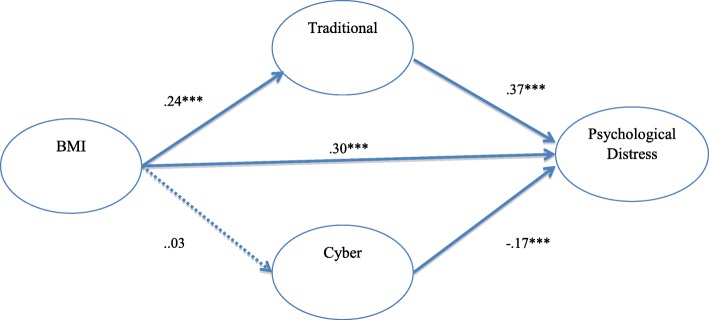
SEM: Direct and Indirect Effects of BMI on Psychological Distress; The figure shows that the proportion of total “BMI” effect mediated via “Traditional” and “Cyber” Victimization was .27. Circles represent observed variables, and straight arrows connect the observed variables. Bold lines represent significant paths, and dotted lines represent nonsignificant paths. All significant parameters are significant at the *p* < .001 level

As can be seen in Figs. 1 and 2, the test of mediation showed a significant indirect effect of BMI on psychological distress through victimization of both traditional and cyber bullying (total indirect = .08; total direct = .30; z = 6.81; *p* < .01) and these indirect effects accounted for 27% of the effect of BMI on psychological distress. As illustrated in Figs. 3 and 4, the results also showed that the indirect effects of traditional and cyber bullying victimization were significant; therefore, bullying victimization significantly mediated the association between BMI and physical distress (total indirect = .03; total direct = .19; z = 7.47; p < .01), while the model explained 16% of variance in physical distress.

## Discussion

A number of findings emerged from the current study. First, obesity, measured by BMI, showed a significant direct effect on one of the two types of victimization –traditional bullying. In line with prior research [26–28, 65, 66], obese or overweight youth are significantly more likely to be victimized by bullying compared to those who are not obese. Contrary to the earlier findings suggesting a positive association between obesity and cyberbullying victimization [28, 29], there was not a significant effect of BMI on the probability of being bullied on-line.

In light of previous research documenting the effect of obesity on physical and psychological distress, statistical evidence was found for a significant link between BMI and both forms of distress. In general, prior obesity research shows that children with obesity had a greater likelihood of exhibiting poor physical and psychological health outcomes [2, 7, 8, 12, 13, 67]. Regarding the impact of victimization on distress, traditional bullying victimization was positively linked to physical and psychological forms of distress. This is consistent with previous research and suggests that youth who have been victims of traditional bullying are more likely to experience a variety of physical and psychological symptoms [33–35]. Yet, an unexpected finding is the negative effect of cyberbullying victimization on psychological distress. Unlike prior studies [36, 40, 41], our study found that youth who have been a victim of cyberbullying are less likely to experience psychosocial distress. One possible explanation may be that individuals may be involved as both victims and perpetrators of cyberbullying and also engage in aggressive behavior as a coping or defense strategy [68–70]. This may contribute to lower levels of psychological distress. Moreover, youth with high levels of self-control showed greater levels of resiliency and lower levels of distress in response to real world or cyberbullying [71]. Finally, peers may serve as a protective role in buffering the negative link between cyberbullying victimization and distress [72].

Notably, mediating effects of bullying victimization were observed using the SUR approach. Only traditional bullying victimization mediated the link between BMI and physical distress. In addition, the association between BMI and psychological distress among youth was mediated by both forms of victimization. While traditional bullying victimization had a positive mediating effect on the BMI-distress link, cyberbullying victimization indicated a negative effect in the analysis. These indirect effects imply that obese or overweight youth who have been victims of traditional bullying would justifiably experience a higher level of physical and psychological distress. Overall, these results offer evidence that there may be further mediating link between BMI, bullying victimization and distress, which warrants further exploration.

The negative mediating effect of cyber victimization on the association between BMI and psychological distress could be explained in several ways. First, the measures for cyberbullying victimization may not fully capture the intricacies of how technology could be subverted to damage a victim’s reputation, self-esteem, or friendship. Rather, it merely reflects the location of bullying using a computer or cell phone. Moreover, victimization may not necessarily lead to emotional distress if it takes place in virtual realm [73]. Given the unique properties of online environments [74], it is plausible that obese or overweight victims may receive social support from bystanders via social media (e.g. Facebook), which, in turn, could neutralize negative comments from peers during weight-based cyberbullying incidents [31]. For instance, if a youth receive a demeaning message or image related to obesity or overweight, an empathetic bystander could dissent (rather than conform to) the negative content made by others, which may be accompanied by reduced distress. Finally, the children in the current sample may not be considered “pure” victims. Since physical dominance is less visible in on-line interactions, it is possible that victims of traditional bullying could engage in aggressive behaviors towards those who have bullied them via electronic means in seeking retribution [75, 76].

### Strengths and limitations

The current study has a number of limitations. First, the results do not allow to draw causal inferences due to the cross-sectional nature of this study. Longitudinal studies are needed to disentangle the temporal relationship between the variables under study. Second, the measure for cyberbullying victimization may not accurately represent the ways in which an individual may be harassed or bullied via the Internet. Cyberbullying could be facilitated via a wide range of on-line platforms such as chat rooms, emails, text messages, mobile phone call, photo or video clip, and social media [77–79]. Future research should consider a more comprehensive measure of cyber victimization to allow for a thorough evaluation of the on-line victimization experience. At the same time, there is need to establish a commonly agreed definition of cyber victimization for ensuring reliability to some degree [80]. In addition, since the current study focused only on victims of weight-based bulling, future research could benefit from exploring the subgroup of bully-victims, who have been found to be more common in cyberbullying [81] as opposed to traditional bullying [82, 83]. Lastly, the present study utilized self-reported questionnaire and hence is subject to response bias.

Our study offers several strengths. First, the dataset used in this study consists of a nationally representative sample of U.S. youth, which enhances generalizability and statistical power and lessens selection bias. Second, despite its deficiency, BMI is considered to be well-validated and widely used by obesity researchers, and further proven to be of good predictive value [84–86]. Third, the use of seemingly unrelated regression (SUR) and Sobel-Goodman tests allows one to assess the extent and significance of the mediating effects of traditional and cyber victimization in the link between weight status and both types of distress. Furthermore, the present findings offer useful insights into the mechanism indirectly linking indicators of obesity to physical and psychological distress via victimization. Yet, future research is needed to untangle the impact of on-line victimization on the link between obesity and psychological distress.

## Conclusions

The primary aim of this study was to examine the direct and indirect effect of obesity on two forms of distress (physical and psychological) among U.S. youth. Our findings affirm that obese and overweight adolescents are more likely to be victims of traditional victimization and also more likely to experience physical and psychological distress compared to those with healthy weight. As an additional finding, victimization can exacerbate the effect of obesity on the level of distress, suggesting traditional victimization as an important mediator in the association between obesity and physical/psychological distress. The current findings underscore the need to raise awareness for the detrimental impact of victimization occurring off-line in school classrooms or playgrounds on the physical and psychological distress among obese and overweight youth. Bullying victimization represents a risk factor for youths’ psychosocial well-being. Parents and school administrators can develop educational interventions to raise the awareness on weight stigma and stereotypes as well as adverse consequences of weight-based bullying. Given that weight-related stigma and teasing occur in school settings among peers, school-based interventions can be implemented to reduce the stigma and bias associated with obesity and overweight and address the ways in which families and communities can come together to educate children to be tolerant of differences in weight and body size. In line with this, youths’ attitudes toward body image have been found to be influenced by social norms [87]. Efforts to reduce weight stigma and discrimination should focus on classroom instructions to improve adolescents’ attitudes toward peers with obesity, school policies prohibiting weight-based bullying, and programs to promote an environment that recognizes and supports the diversity of cultural foodways [88]. Hence, a comprehensive school-wide approach is necessary to adequately address the ongoing challenges that are faced in the prevention of weight-based victimization among youth.

## Abbreviations

ADHD: Attention Deficit Hyperactivity Disorder
BMI: Body Mass Index
CFI: Comparative Fit Index
RMSEA: Root Mean Square Error of Approximation
SD: Standard Deviation
SEM: Structural Equation Modeling
SRMR: Standardized Root Mean Square Residual
SUR: Seemingly Unrelated Regression

## Footnotes

1 Participants were given a definition of “being bullied” as follows: *A student is being bullied when another student, or a group of students, say or do nasty or unpleasant things to him or her. It is also bullying when a student is teased repeatedly in a way he or she does not like or when they are deliberately left out of things. But it is not bullying when two students of about the same strength or power argue or fight. It is also not bullying when a student is teased in a friendly and playful way.*

2 According to the Center for Disease Control’s BMI category, the raw BMI score for boys and girls is different. However, the CDC adjust raw BMI score into BMI categories based upon centralized-percentiles cutoffs regardless of participants’ sex. (See BMI-for-age charts). Therefore, the interpretation of BMI is not different.
